# 4,6-Dinitro­pyrogallol

**DOI:** 10.1107/S1600536812004771

**Published:** 2012-02-17

**Authors:** Christian Neis, Günter J. Merten, Kaspar Hegetschweiler

**Affiliations:** a Fachrichtung Chemie, Universität des Saarlandes, Postfach 151150, D-66041 Saarbrücken, Germany

## Abstract

In the title mol­ecule, C_6_H_4_N_2_O_7_, the two nitro groups are tilted with respect to the aromatic ring by 11.2 (1) and 10.9 (1)°. All three hy­droxy groups are involved in the formation of bifurcated intra- and inter­molecular O—H⋯O hydrogen bonds. The crystal packing exhibits short O⋯O distances of 2.823 (2) Å between two O atoms of the nitro groups.

## Related literature

The synthesis of the title compound has recently been reported by Merten *et al.* (2012 ▶). The importance of hydrogen bonding, π stacking and donor–acceptor inter­actions in solid-state structures and in solution has been reviewed by Schneider (2009 ▶). The crystal structure of dinitro­phloroglucinol, a constitutional isomer of the title compound, has been described by Schweitzer *et al.* (2008 ▶). H atoms were treated as recommended by Müller *et al.* (2006 ▶).
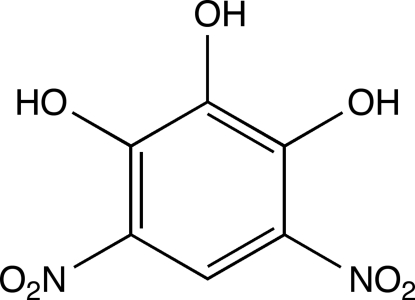


## Experimental

### 

#### Crystal data

C_6_H_4_N_2_O_7_
*M* *_r_* = 216.11
Monoclinic, 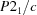

*a* = 6.7612 (14) Å
*b* = 10.878 (2) Å
*c* = 10.297 (2) Å
β = 92.75 (3)°
*V* = 756.5 (3) Å^3^
*Z* = 4
Mo *K*α radiation
μ = 0.18 mm^−1^
*T* = 200 K
0.60 × 0.22 × 0.20 mm

#### Data collection

Stoe IPDS image plate diffractometer
5824 measured reflections
1488 independent reflections
1326 reflections with *I* > 2σ(*I*)
*R*_int_ = 0.059

#### Refinement

*R*[*F* ^2^ > 2σ(*F* ^2^)] = 0.031
*wR*(*F* ^2^) = 0.089
*S* = 1.06
1488 reflections
146 parameters
3 restraints
H atoms treated by a mixture of independent and constrained refinement
Δρ_max_ = 0.31 e Å^−3^
Δρ_min_ = −0.23 e Å^−3^

### 

Data collection: *IPDS Software* (Stoe & Cie, 1997 ▶); cell refinement: *IPDS Software*; data reduction: *IPDS Software*; program(s) used to solve structure: *SHELXS97* (Sheldrick, 2008 ▶); program(s) used to refine structure: *SHELXL97* (Sheldrick, 2008 ▶); molecular graphics: *DIAMOND* (Brandenburg, 2011 ▶); software used to prepare material for publication: *SHELXL97*.

## Supplementary Material

Crystal structure: contains datablock(s) global, I. DOI: 10.1107/S1600536812004771/cv5241sup1.cif

Structure factors: contains datablock(s) I. DOI: 10.1107/S1600536812004771/cv5241Isup2.hkl

Supplementary material file. DOI: 10.1107/S1600536812004771/cv5241Isup3.cml

Additional supplementary materials:  crystallographic information; 3D view; checkCIF report

## Figures and Tables

**Table 1:** Hydrogen-bond geometry (Å, °)

*D*—H⋯*A*	*D*—H	H⋯*A*	*D*⋯*A*	*D*—H⋯*A*
O2—H2O⋯O3	0.85 (1)	2.14 (2)	2.6467 (14)	118 (2)
O2—H2O⋯O4^i^	0.85 (1)	2.27 (2)	2.9531 (14)	138 (2)
O1—H1O⋯O6	0.86 (2)	1.91 (2)	2.6359 (15)	141 (2)
O1—H1O⋯O6^ii^	0.86 (2)	2.46 (2)	3.1361 (15)	137 (2)
O3—H3O⋯O4	0.83 (2)	1.97 (2)	2.6357 (14)	137 (2)
O3—H3O⋯O7^iii^	0.83 (2)	2.19 (2)	2.7658 (15)	127 (2)

Symmetry codes: (i) 
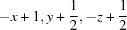
; (ii) 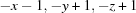; (iii) 
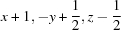
.

**Fig. 1.**
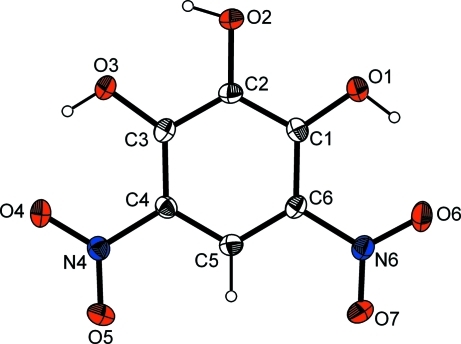
Molecular structure of the title compound. Displacement ellipsoids are drawn at the 50% probability level.

**Fig. 2.**
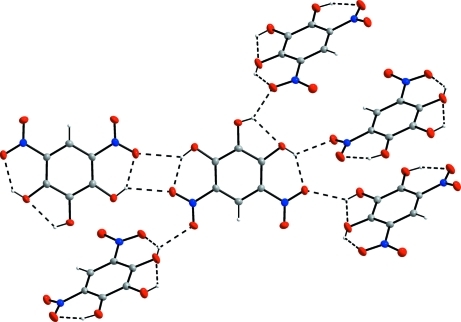
Section of the hydrogen bonding network, showing bifurcated hydrogen bonds for all three hydroxy groups (displacement ellipsoids are drawn at the 50% probability level).

## supplementary crystallographic information

### Comment

As expected, the aromatic C_6_-ring is planar (the positions of the six C atoms deviate from the mean plane by 0.0025 (8)–0.0196 (8) Å). The C_6_-ring and the two nitro groups are also approximately coplanar, allowing significant π-delocalization. In the extended hydrogen bonding network all three phenolic hydroxy groups act as hydrogen donors and the nitro groups as hydrogen acceptors. The O atoms O1 and O6 of two neighbouring molecules form a planar, four-membered O1···O6···O1'···O6' ring with two bifurcated hydrogen bonds. Remarkably, the O6···O6' distance of 2.823 (2) Å (*i.e.* one of the diagonal of the corresponding parallelogram) is somewhat shorter than the sum of the van der Waals radii, although no direct hydrogen bond between these two atoms is operative. Some intermolecular C···C and C···O distances (C1···C1': 3.279 Å, C5···O2": 3.046 Å) are slightly shorter than the sum of the van der Waals radii and may be interpreted in terms of π-stacking or weak donor–acceptor interactions.

### Experimental

The title compound has been prepared by nitration of pyrogallol-triacetate. ^1^H NMR ([D_6_]acetone): δ (p.p.m.) = 8.51. ^13^C NMR ([D_6_]acetone): δ (p.p.m.) = 114.4, 128.7, 136.6, 148.8. Elemental analysis calculated for C_6_H_4_N_2_O_7_ (%): C 33.35, H 1.87, N 12.96; found (%): C 33.19, H 2.24, N 12.83. Single crystals were obtained directly from the reaction mixture at 278 K.

### Refinement

All non-hydrogen atoms were refined using anisotropic displacement parameters. H atoms were treated as recommended by Müller *et al.* (2006). A riding model was used for the C-bonded H5. The positional parameters of the O-bonded H1O, H2O and H3O were refined using isotropic displacement parameters which were set to 1.5*U*_eq_ of the pivot atom. In addition, a restraint of 0.84 Å was used for the O—H distances.

### Crystal data

**Table:**

C_6_H_4_N_2_O_7_	*Z* = 4
*M**_r_* = 216.11	*F*(000) = 440
Monoclinic, *P*2_1_/*c*	*D*_x_ = 1.898 Mg m^−^^3^
*a* = 6.7612 (14) Å	Mo *K*α radiation, λ = 0.71073 Å
*b* = 10.878 (2) Å	µ = 0.18 mm^−^^1^
*c* = 10.297 (2) Å	*T* = 200 K
β = 92.75 (3)°	Prism, light brown
*V* = 756.5 (3) Å^3^	0.60 × 0.22 × 0.20 mm

### Data collection

**Table:**

Stoe IPDS image plate diffractometer	1326 reflections with *I* > 2σ(*I*)
Radiation source: fine-focus sealed tube	*R*_int_ = 0.059
Graphite monochromator	θ_max_ = 26.0°, θ_min_ = 2.7°
φ scans	*h* = −8→8
5824 measured reflections	*k* = −13→13
1488 independent reflections	*l* = −12→12

### Refinement

**Table:**

Refinement on *F*^2^	Secondary atom site location: difference Fourier map
Least-squares matrix: full	Hydrogen site location: inferred from neighbouring sites
*R*[*F*^2^ > 2σ(*F*^2^)] = 0.031	H atoms treated by a mixture of independent and constrained refinement
*wR*(*F*^2^) = 0.089	*w* = 1/[σ^2^(*F*_o_^2^) + (0.0484*P*)^2^ + 0.1574*P*] where *P* = (*F*_o_^2^ + 2*F*_c_^2^)/3
*S* = 1.06	(Δ/σ)_max_ < 0.001
1488 reflections	Δρ_max_ = 0.31 e Å^−^^3^
146 parameters	Δρ_min_ = −0.23 e Å^−^^3^
3 restraints	Extinction correction: *SHELXL97* (Sheldrick, 2008), Fc^*^=kFc[1+0.001xFc^2^λ^3^/sin(2θ)]^-1/4^
Primary atom site location: structure-invariant direct methods	Extinction coefficient: 0.093 (9)

### Special details

**Table:**

Geometry. All e.s.d.'s (except the e.s.d. in the dihedral angle between two l.s. planes) are estimated using the full covariance matrix. The cell e.s.d.'s are taken into account individually in the estimation of e.s.d.'s in distances, angles and torsion angles; correlations between e.s.d.'s in cell parameters are only used when they are defined by crystal symmetry. An approximate (isotropic) treatment of cell e.s.d.'s is used for estimating e.s.d.'s involving l.s. planes.
Refinement. Refinement of *F*^2^ against ALL reflections. The weighted *R*-factor *wR* and goodness of fit *S* are based on *F*^2^, conventional *R*-factors *R* are based on *F*, with *F* set to zero for negative *F*^2^. The threshold expression of *F*^2^ > σ(*F*^2^) is used only for calculating *R*-factors(gt) *etc*. and is not relevant to the choice of reflections for refinement. *R*-factors based on *F*^2^ are statistically about twice as large as those based on *F*, and *R*-factors based on ALL data will be even larger.

### Fractional atomic coordinates and isotropic or equivalent isotropic displacement parameters (Å^2^)

**Table:**

	*x*	*y*	*z*	*U*_iso_*/*U*_eq_	
C5	0.03683 (18)	0.22251 (11)	0.46470 (11)	0.0173 (3)	
H5	0.0181	0.1542	0.5203	0.021*	
C2	0.09313 (18)	0.42549 (12)	0.30137 (11)	0.0176 (3)	
C6	−0.10033 (17)	0.31771 (12)	0.45731 (11)	0.0166 (3)	
C1	−0.07860 (17)	0.42018 (11)	0.37270 (11)	0.0168 (3)	
C4	0.20152 (17)	0.22875 (11)	0.38968 (11)	0.0167 (3)	
C3	0.23403 (17)	0.33087 (12)	0.30757 (11)	0.0176 (3)	
O2	0.11938 (14)	0.52556 (9)	0.22378 (9)	0.0253 (3)	
H2O	0.235 (2)	0.5179 (17)	0.1964 (17)	0.038*	
O1	−0.20607 (13)	0.51466 (9)	0.35774 (9)	0.0228 (3)	
H1O	−0.310 (2)	0.4983 (17)	0.3996 (17)	0.034*	
O3	0.38713 (14)	0.34789 (10)	0.23055 (10)	0.0265 (3)	
H3O	0.467 (3)	0.2905 (15)	0.2362 (18)	0.040*	
N4	0.34037 (15)	0.12578 (10)	0.39485 (10)	0.0184 (3)	
N6	−0.26572 (15)	0.31109 (10)	0.54231 (10)	0.0195 (3)	
O4	0.50228 (13)	0.14010 (9)	0.34200 (9)	0.0250 (3)	
O5	0.29368 (14)	0.03088 (9)	0.44938 (9)	0.0259 (3)	
O6	−0.40392 (14)	0.38686 (10)	0.52605 (9)	0.0288 (3)	
O7	−0.26491 (15)	0.23213 (9)	0.62784 (10)	0.0289 (3)	

### Atomic displacement parameters (Å^2^)

**Table:**

	*U*^11^	*U*^22^	*U*^33^	*U*^12^	*U*^13^	*U*^23^
C5	0.0202 (6)	0.0161 (6)	0.0156 (5)	−0.0017 (5)	0.0016 (4)	−0.0004 (5)
C2	0.0208 (6)	0.0168 (6)	0.0152 (6)	−0.0010 (5)	0.0011 (4)	0.0022 (4)
C6	0.0159 (6)	0.0189 (6)	0.0154 (6)	−0.0021 (5)	0.0029 (4)	−0.0024 (4)
C1	0.0179 (6)	0.0171 (6)	0.0151 (6)	0.0020 (5)	−0.0010 (4)	−0.0027 (4)
C4	0.0189 (6)	0.0150 (6)	0.0162 (6)	0.0017 (4)	0.0007 (5)	−0.0014 (4)
C3	0.0165 (6)	0.0208 (6)	0.0158 (6)	−0.0009 (5)	0.0025 (4)	−0.0013 (5)
O2	0.0246 (5)	0.0238 (5)	0.0283 (5)	0.0039 (4)	0.0084 (4)	0.0113 (4)
O1	0.0209 (5)	0.0231 (5)	0.0247 (5)	0.0065 (4)	0.0048 (4)	0.0036 (4)
O3	0.0227 (5)	0.0280 (6)	0.0300 (5)	0.0065 (4)	0.0135 (4)	0.0090 (4)
N4	0.0199 (5)	0.0191 (6)	0.0163 (5)	0.0019 (4)	0.0011 (4)	−0.0012 (4)
N6	0.0189 (5)	0.0196 (6)	0.0204 (5)	−0.0016 (4)	0.0041 (4)	−0.0027 (4)
O4	0.0208 (5)	0.0265 (5)	0.0284 (5)	0.0056 (4)	0.0084 (4)	0.0003 (4)
O5	0.0306 (5)	0.0186 (5)	0.0288 (5)	0.0030 (4)	0.0040 (4)	0.0062 (4)
O6	0.0214 (5)	0.0345 (6)	0.0314 (5)	0.0085 (4)	0.0091 (4)	0.0023 (4)
O7	0.0319 (5)	0.0252 (5)	0.0310 (5)	0.0009 (4)	0.0155 (4)	0.0071 (4)

### Geometric parameters (Å, º)

**Table:**

C5—C4	1.3870 (17)	C4—N4	1.4609 (16)
C5—C6	1.3898 (17)	C3—O3	1.3467 (15)
C5—H5	0.9500	O2—H2O	0.846 (14)
C2—O2	1.3669 (15)	O1—H1O	0.859 (15)
C2—C3	1.4019 (18)	O3—H3O	0.827 (15)
C2—C1	1.4046 (17)	N4—O5	1.2237 (15)
C6—C1	1.4267 (17)	N4—O4	1.2554 (14)
C6—N6	1.4545 (15)	N6—O7	1.2301 (15)
C1—O1	1.3454 (15)	N6—O6	1.2512 (15)
C4—C3	1.4195 (18)		
			
C4—C5—C6	118.97 (11)	C5—C4—N4	118.28 (11)
C4—C5—H5	120.5	O3—C3—C2	114.34 (11)
C6—C5—H5	120.5	O3—C3—C4	127.14 (12)
O2—C2—C3	120.29 (11)	C2—C3—C4	118.51 (11)
O2—C2—C1	118.18 (11)	C2—O2—H2O	105.3 (13)
C3—C2—C1	121.53 (11)	C1—O1—H1O	108.5 (13)
C5—C6—C1	121.85 (11)	C3—O3—H3O	111.9 (13)
C5—C6—N6	117.42 (11)	O5—N4—O4	123.58 (11)
C1—C6—N6	120.71 (11)	O5—N4—C4	118.95 (11)
O1—C1—C2	116.73 (11)	O4—N4—C4	117.47 (10)
O1—C1—C6	125.64 (11)	O7—N6—O6	122.24 (11)
C2—C1—C6	117.61 (11)	O7—N6—C6	119.22 (11)
C5—C4—C3	121.43 (11)	O6—N6—C6	118.54 (10)
			
C4—C5—C6—C1	1.40 (18)	O2—C2—C3—C4	179.79 (11)
C4—C5—C6—N6	−176.90 (10)	C1—C2—C3—C4	−0.88 (18)
O2—C2—C1—O1	0.65 (16)	C5—C4—C3—O3	179.92 (11)
C3—C2—C1—O1	−178.70 (11)	N4—C4—C3—O3	−1.37 (19)
O2—C2—C1—C6	−177.52 (10)	C5—C4—C3—C2	−1.28 (18)
C3—C2—C1—C6	3.13 (17)	N4—C4—C3—C2	177.43 (10)
C5—C6—C1—O1	178.59 (11)	C5—C4—N4—O5	10.41 (16)
N6—C6—C1—O1	−3.17 (18)	C3—C4—N4—O5	−168.34 (11)
C5—C6—C1—C2	−3.43 (17)	C5—C4—N4—O4	−169.81 (10)
N6—C6—C1—C2	174.81 (10)	C3—C4—N4—O4	11.44 (16)
C6—C5—C4—C3	1.01 (18)	C5—C6—N6—O7	9.35 (16)
C6—C5—C4—N4	−177.72 (10)	C1—C6—N6—O7	−168.96 (11)
O2—C2—C3—O3	−1.27 (17)	C5—C6—N6—O6	−171.03 (10)
C1—C2—C3—O3	178.07 (10)	C1—C6—N6—O6	10.65 (16)

### Hydrogen-bond geometry (Å, º)

**Table:**

*D*—H···*A*	*D*—H	H···*A*	*D*···*A*	*D*—H···*A*
O2—H2*O*···O3	0.85 (1)	2.14 (2)	2.6467 (14)	118 (2)
O2—H2*O*···O4^i^	0.85 (1)	2.27 (2)	2.9531 (14)	138 (2)
O1—H1*O*···O6	0.86 (2)	1.91 (2)	2.6359 (15)	141 (2)
O1—H1*O*···O6^ii^	0.86 (2)	2.46 (2)	3.1361 (15)	137 (2)
O3—H3*O*···O4	0.83 (2)	1.97 (2)	2.6357 (14)	137 (2)
O3—H3*O*···O7^iii^	0.83 (2)	2.19 (2)	2.7658 (15)	127 (2)

Symmetry codes: (i) −*x*+1, *y*+1/2, −*z*+1/2; (ii) −*x*−1, −*y*+1, −*z*+1; (iii) *x*+1, −*y*+1/2, *z*−1/2.
